# The value of glycosylated hemoglobin in the diagnosis of diabetic retinopathy: a systematic review and Meta-analysis

**DOI:** 10.1186/s12902-021-00737-2

**Published:** 2021-04-26

**Authors:** Bo Zhang, Bingjie Zhang, Zhulin Zhou, Yutong Guo, Dan Wang

**Affiliations:** 1 Department of Neurosurgery, First Hospital of Jilin University, No. 1 Xinmin Street, Changchun, 130021 Jilin China; 2 Department of Ophthalmology, First Hospital of Jilin University, No. 1 Xinmin Street, Changchun, 130021 Jilin China; 3 Department of Neurobiology, Care Sciences & Society, Division of Neurogeriatrics, Karolinska Institutet, Karolinska University Hospital Solna, Stockholm, Sweden; 4 St. Erik Eye hospital, Stockholm, Sweden

**Keywords:** Glycosylated hemoglobin, Diagnosis, Diabetic retinopathy, Cut-off value, Meta-analysis

## Abstract

**Objective:**

Glycosylated hemoglobin (HbA1c) has obvious clinical value in the diagnosis of diabetes, but the conclusions on the diagnostic value of diabetic retinopathy (DR) are not consistent. This study aims to comprehensively evaluate the accuracy of glycosylated hemoglobin in the diagnosis of diabetic retinopathy through the meta-analysis of diagnostic tests.

**Methods:**

Cochrane Library, Embase, PubMed, Web of Science, China National Knowledge Infrastructure (CNKI), China Wanfang Database, Chinese Biomedical Literature Database (CBM) were searched until November, 2020. The Quality Assessment of Diagnostic Accuracy Studies-2 (QUADAS-2) tool was used to assess the quality of the included studies. The pooled sensitivity, specificity, positive likelihood ratio (+LR), negative likelihood ratio (-LR), diagnostic odds ratio (DOR) and areas under the receiver operating characteristic (ROC) curve were calculated by Stata 15.0 software.

**Results:**

After screening, 18 high-quality papers were included. The results of meta-analysis showed that the combined DOR = 18.19 (95% CI: 10.99–30.11), the sensitivity= 0.81 (95% CI): 0.75 ~ 0.87), specificity = 0.81 (95%CI: 0.72 ~ 0.87), +LR = 4.2 (95%CI: 2.95 ~ 6.00), −LR = 0.23 (95%CI: 0.17 ~ 0.31), and the area under the Summary ROC curve was 0.88 (95%CI:  0.85 ~ 0.90).

**Conclusion:**

The overall accuracy of HbA1cC forin diagnosing diabetic retinopathy is good. As it is more stable than blood sugar and is not affected by meals, it may be a suitable indicator for diabetic retinopathy.

## Introduction

 Diabetes mellitus (DM) is a global pandemic. According to statistics, the number of diabetes in the world reached 366 million in 2011 and 422 million in 2014 (accounting for 8.5% of the population). The International Diabetes Federation predicts [1] that the number of diabetes will rise to 552 million by 2030, of which type 2 diabetes mellitus (T2DM) will accounts for 90%, while the situation in developing countries will be even more severe. According to the current clinical disease and case statistics, diabetic retinopathy is not only one of the serious complications of diabetes, but also the main cause of blindness in adults. A recent analysis report shows that about 93 million people (35%) of diabetic patients worldwide have diabetic retinopathy (DR), and 28 million (10%) of whose vision has already been affected [2]. Among patients with type 2 diabetes, an average of about 50% of them will develop diabetic retinopathy, 20 years after the onset of the disease, and about 10% will have proliferative diabetic retinopathy or exudative macular degeneration [3]. According to statistics, among the patients with diabetes in China, the prevalence rate of diabetic retinopathy is close to 40%. With the prolongation of the course of diabetes, the prevalence rate of diabetic retinopathy has increased to 54% [4]. A number of foreign studies have shown that among patients with type 2 diabetes, the possibility of diabetic retinopathy increases year by year with the continuous prolongation of the course of disease. About 30 to 60% of patients with diabetes will develop DR [5, 6]. The occurrence of diabetic retinopathy not only affects people’s life to a great extent, but also affects people’s quality of life, which increases the social and economic pressure of the country and patients’ families as well as patients themselves [7].It has also been found that retinopathy in patients with type 2 diabetes is not diagnosed until at least 7 years after patients get retinopathy. Due to the slow onset of diabetic retinopathy, if the disease does not invade the macula, it is not easy to be diagnosed until the patient comes to see a doctor with symptoms such as blurred vision and decreased vision. At this time, the disease has developed to the stage of irreversible and severe microangiopathy, when it has well past the best period of treatment, so it is very difficult to treat it, and the treatment effect is relatively poor, which eventually results in patients’ blindness. Therefore, it is imperative to detect and treat the risk factors that affect the disease in the early stage.

Studies have shown that a variety of risk factors are closely related to the occurrence and development of diabetic retinopathy, such as hyperglycemia, course of disease [8], hypertension, dyslipidemia and so on [9, 10]. United Kingdom Prospective Diabetes Study (UKPDS) and Diabetes Control and Complications Trial (DCCT) have been followed up for 10 years, showing that early and continuous hyperglycemia control is beneficial for improving diabetic retinopathy [11, 12]. In addition, studies have shown that for every 1% reduction in glycosylated hemoglobin, the risk of microvascular complications (mainly diabetic retinopathy) can be reduced by 37% [13]. For many years, the relationship between hyperglycemia and DR has been studied mainly through the determination of glycosylated hemoglobin and / or fasting plasma glucose [14] and other metabolic indexes. Glycosylated hemoglobin (HbA1c) is the product of the combination of hemoglobin and blood sugar. Its concentration in blood is stable and is not affected by short-term blood glucose concentration. It can effectively reflect the level of glucose metabolism in only 3 months. At present, it is internationally recognized as the gold standard for long-term blood glucose control [15]. Some studies have shown that the occurrence and development of DR is closely related to the level of HbA1c [16].

In order to objectively evaluate the diagnostic value of HbA1c in diabetic retinopathy, this study used the meta-analysis method to systematically analyze the published tests of HbA1c in the diagnosis of diabetic retinopathy home and abroad, so as to provide reference for clinical application in the future.

## Methods

### Retrieval strategy

Two researchers independently searched Cochrane Library, Embase, PubMed, Web of Science, China Knowledge Network China National Knowledge Infrastructure (CNKI), China Wanfang Database, Chinese Biomedical Literature Database (CBM) from inception to November 2020 to evaluate the value of HbA1C in the diagnosis of diabetic retinopathy. The search strategy was as follows: (“HbA1c” OR “HBALc” OR “glycosylated hemoglobin” OR “glycated hemoglobin”) AND (“DR” OR “diabetic retinopathy” ). There was no language limitation. When necessary, we looked for additional references from review articles, guides and conferences.

### Literature selection criteria

#### Inclusion criteria

1) Studies using HbA1c to diagnose diabetic retinopathy; 2) Studies that include a clear sensitivity and specificity, or a four-grid table that can list diagnostic tests through reported data. 3) when it came to studies with the same or overlapping data for the same author, studies with a relevantly recent publication time or a larger number of subjects were selected.

#### Exclusion criteria

1) repeated studies or papers with incomplete document or wrong data; 2) studies that cannot extract effective data indicators; 3)Research on the diagnosis of diabetes by HbA1c; 4) case study with less than 20 samples; 5) the type of papers included basic research, review, conference abstract, etc.

### Quality assessment

The Quality Assessment of Diagnostic Accuracy Studies-2 (QUADAS-2) was adopted to evaluate the quality of all included studies [17]. Two researchers scored the studies separately according to the evaluation form, and if there were differences in the results, a decision was made through discussion to ensure the quality of the included studies.

### Data extraction

All data were independently extracted by two researchers, cross-checked, and they made a decision through discussion when disagreement occurred. The following information was extracted: 1) author, year of publication, country, race and sample size, etc. 2) diagnostic parameters: the cut-off value ofHbA1c for diagnosing diabetic retinopathy and the four-grid table parameters of the diagnostic test: including true positive value (TP), false positive (FP), true negative (TN), false negative value (FN).

### Statistical analysis

Meta-analysis was carried out by Stata15.0 software. The sensitivity, specificity, positive likelihood ratio (+LR), negative likelihood ratio (-LR) and diagnostic odds ratio (DOR) of HbA1c in the diagnosis of diabetic retinopathy were summarized by the bivariate mixed-effects regression model, and heterogeneity was analyzed. Meta-regression and subgroup analysis waswere used to explore the sources of heterogeneity. The summary receiver operatingor characteristic (SROC) curve, was used to calculate the area under the curve (AUC). A funnel plot was drawn to detect the publication bias. At the same time, a sensitivity analysis was used to verify the robustness of the findings. 

## Results

### Literature research, characteristic and quality of studies

**Fig. 1:**
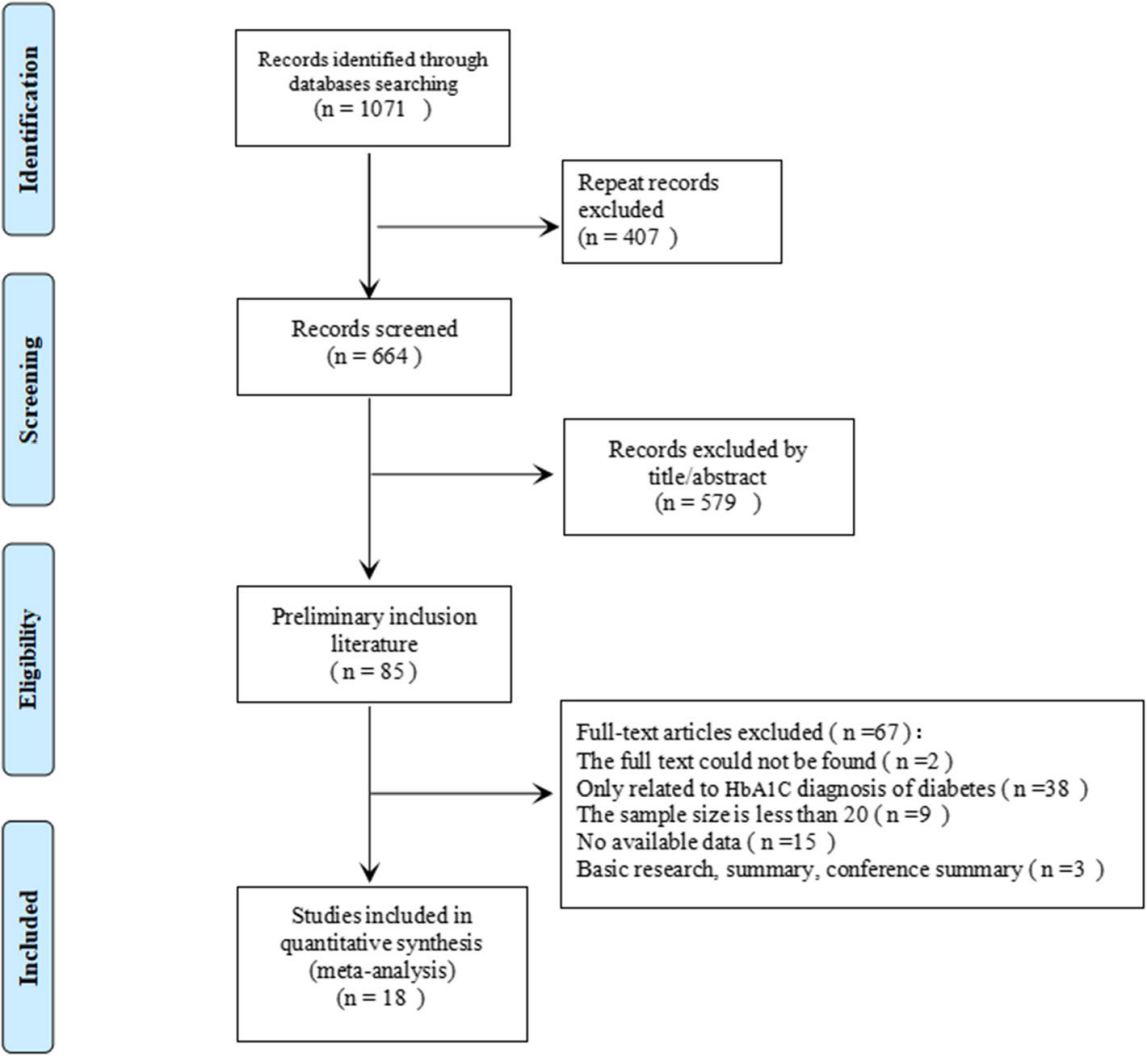
A flow diagram of the study selection process

**Fig. 2:**
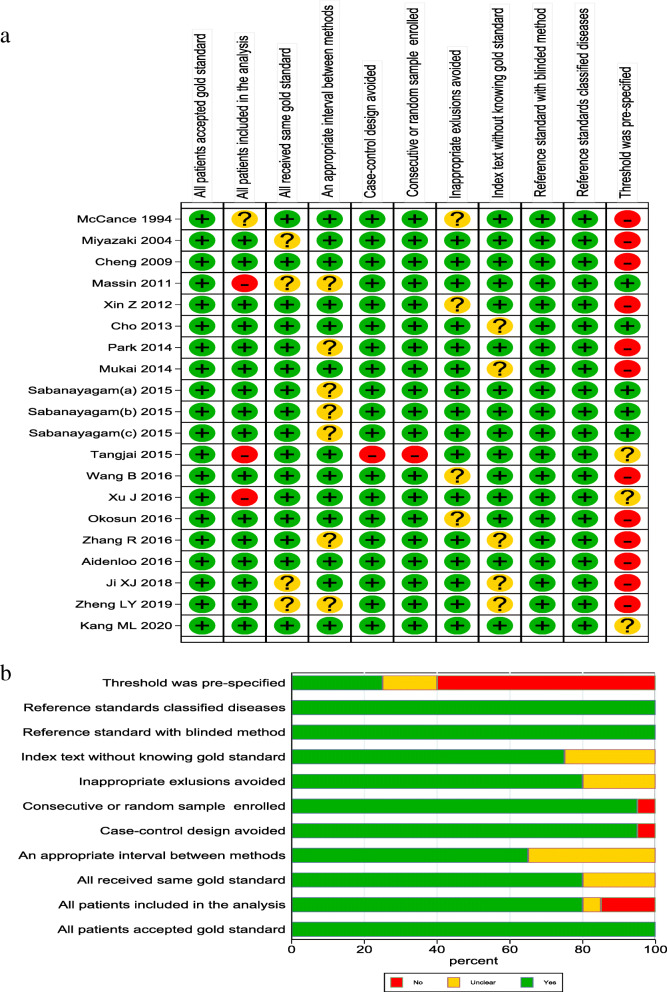
Results of literature quality evaluation according to QUADAS-2. (**a**: Risk of bias summary; **b**: Risk of bias graph)

After preliminary search, a total of 1071 papers were obtained, 407 repeated papers were excluded, 579 papers were removed after reading abstracts, and 67 of them were excluded after further reading the full text. Therefore, this meta-analysis finally included 18 published papers studies [18–35], containing 20 studies.  The screening flow diagram was shown in Fig. 1. For an overview of the included literature, see Table 1. Among the included studies, 7 were from China, 3 from the United States, 2 from South Korea, 2 from Japan, 1 from France, 1 from India, 1 from Thailand and 1 from Iran. The quality of the included literature is shown in Fig. 2 (a, b) The overall quality was high, and only when the threshold was set in advance, there was a high risk of deviation.

**Table 1:** General characteristics of the reviewed studies and the primary results

Study	Year	Country	Number		Age (Years)	Ophthalmic examination test	cut-off value	DR diagnosis			
			DR	Control				TP	FN	FP	TN
McCance [18]	1994	USA	32	927	>25	Direct ophthalmoscopic examination	7.8	21	11	115	812
Miyazaki [19]	2004	Japan	37	1600	40-79	indirect ophthalmoscopy, slit lamp, and 45°fundus photographs.	5.7	32	5	158	1442
Cheng [20]	2009	USA	153	913	≥40	Two 45° nonmydriatic color digital images	5.5	122	21	575	338
Massin [21]	2011	France	44	656	30-65	Three nonmydriatic digital retinal photograph	6	8	36	52	604
Xin Z [22]	2012	China	74	2477	18-79	Two 45°color digital images	6.8	63	11	297	2180
Cho [23]	2013	Korea	63	3340	40-69	single-field nonmydriatic fundus photography	6.7	54	9	528	2812
Park [24]	2014	Korea	115	5097	≥19	45°nonmydriatic digital retina image	6.2	108	7	525	4572
Mukai [25]	2014	Japan	52	2629	40-79	45°fundus photographs	6.1	45	7	294	2335
Sabanayagam (a) [26]	2015	India	165	3575	40-80	two 45°retinal images	6.5	142	23	1004	2571
Sabanayagam (b) [26]	2015	India	137	3459	40-80	two 45°retinal images	6.5	117	20	820	2639
Sabanayagam (c) [26]	2015	India	93	5741	40-80	two 45°retinal images	6.5	70	23	592	5149
Tangjai [27]	2015	Thailand	50	50	45-65	slit lamp	7.25	42	8	17	33
Wang B [28]	2016	China	253	8138	40-90	digital fundus photographs	6.5	204	49	1066	7072
Xu J [29]	2016	China	496	1511	64.1±9.0	45°color digital images	6.5	372	124	855	656
Okosun [30]	2016	USA	93	372	62.6	digital retinal photography	5.2	87	6	290	82
Zhang R [31]	2016	China	40	3084	NR	45°fundus photographs	6.4	25	15	296	2788
Aidenloo [32]	2016	Iran	59	2951	40-81	45°color digital images	6.2	53	3	310	2641
Ji XJ [33]	2018	China	215	285	NR	fundus examination	8.05	149	66	34	251
Zheng LY [34]	2019	China	81	87	42-82	fundus examination	20.01	66	15	33	54
Kang ML [35]	2020	China	93	207	18-87	Ultrasonic	NR	75	18	20	187

*FN* false negative, *FP* false positive, *TN* true negative, *TP* true positive, *NR* not report, *DR* diabetic retinopathy

### The results of the meta-analysis

**Fig. 3:**
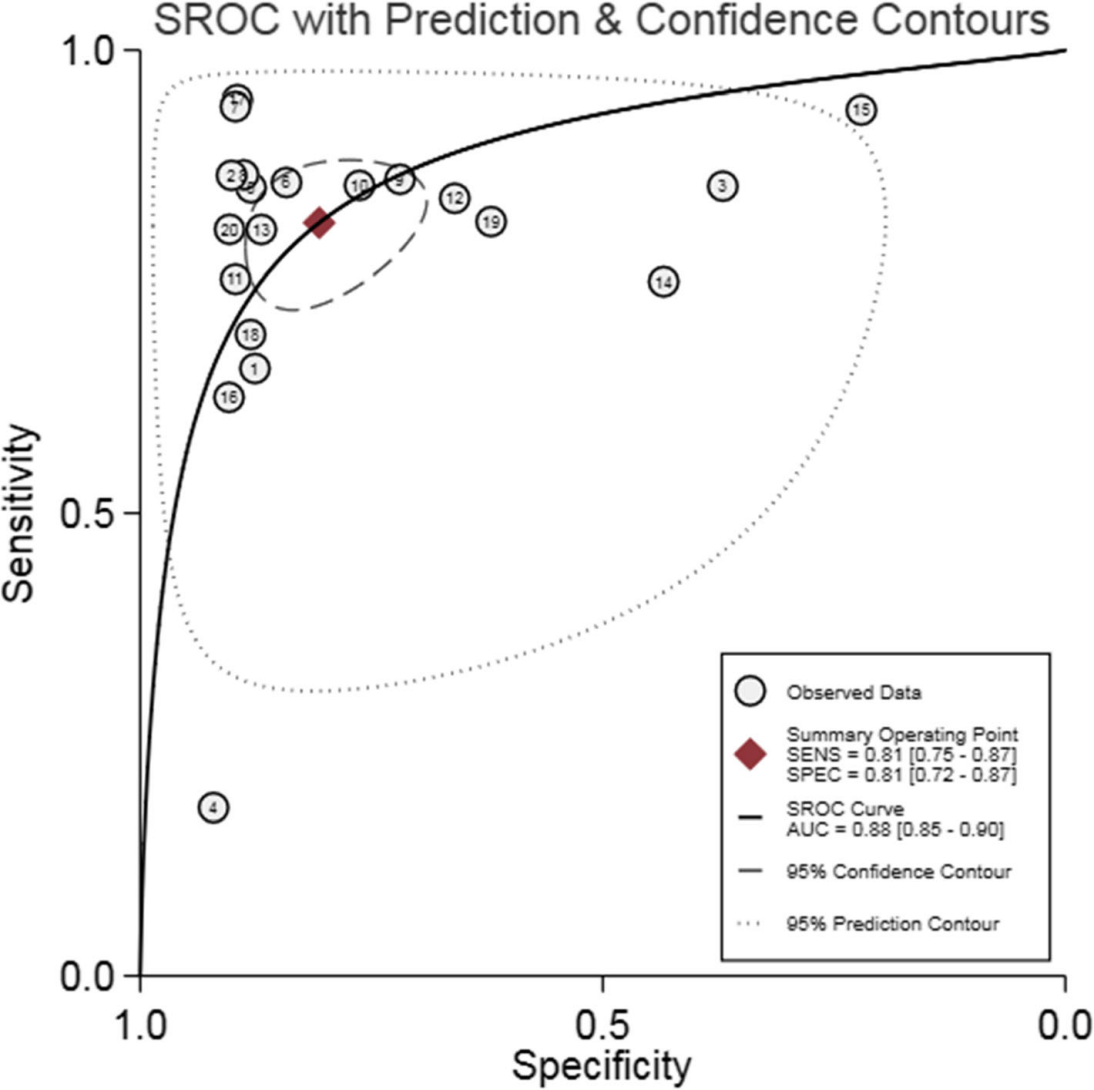
The SROC curve for the accuracy of HbA1c in the diagnosis of diabetic retinopathy. SROC: summary receiver operating characteristic

**Fig. 4:**
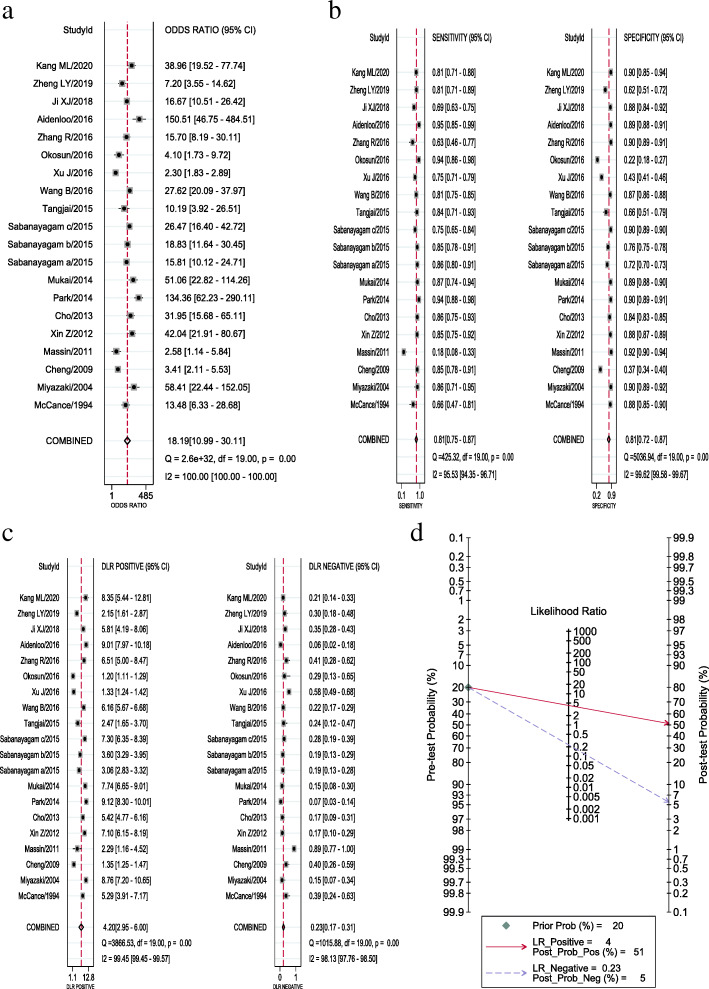
Forest plots of HbA1c in the diagnosis of diabetic retinopathy (**a**: Diagnostic odds ratio; **b**: Sensitivity and specificity; **c**: Positive likelihood ratio and negative likelihood ratio; **d**: Fagan’s Nomogram)

**Fig. 5:**
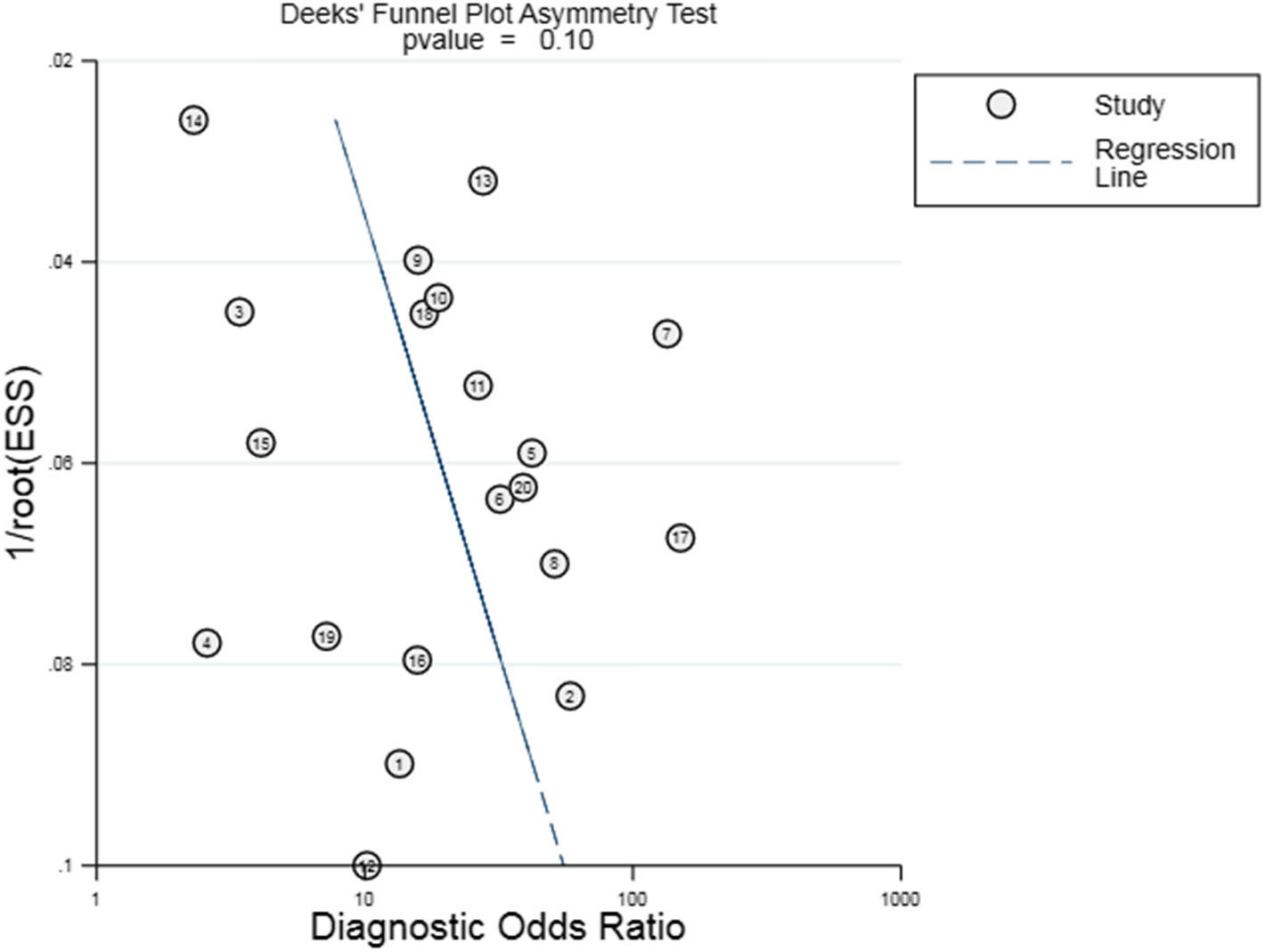
Deeks’ funnel plot of HbA1c for the diagnosis of diabetic retinopathy

**Fig. 6:**
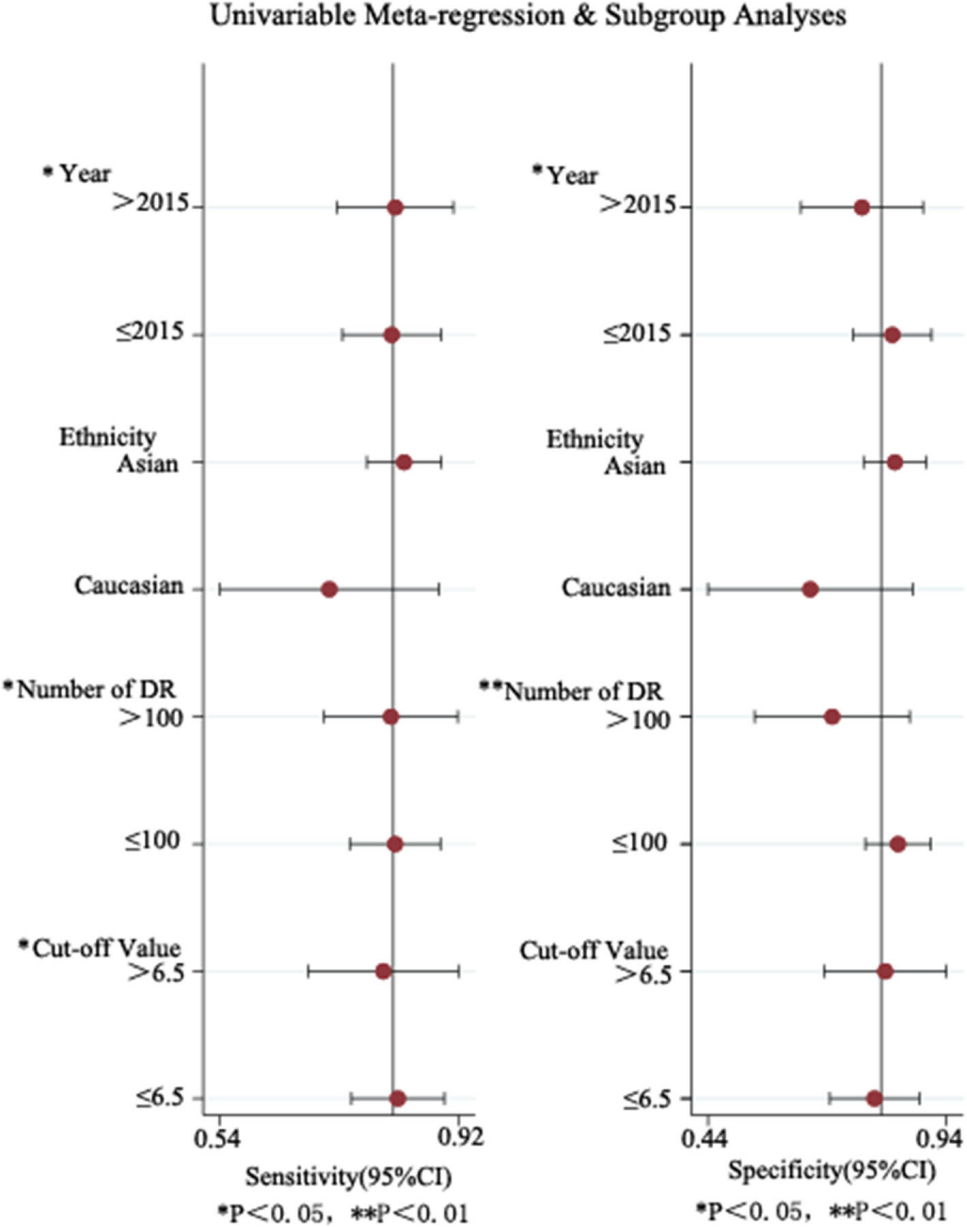
Univariable meta-regression & subgroup analyses of HbA1c for the diagnosis of diabetic retinopathy

In the included papers, the sensitivity logarithm and the (1-specificity) logarithm were analyzed by Spearman correlation, with the correlation coefficient 0.208 (*P* = 0.380) and the SROC curve did not show a typical “shoulder-arm-like” distribution (Fig. 3), indicating that there was no threshold effect in this study. The heterogeneity across the studies was assessed. The DOR (*P* = 0.00, I^2^ = 100.00%), sensitivity (*P* = 0.00, I^2^ = 95.53%), specificity (*P* = 0.00, I^2^ = 99.62%), +LR (*P* = 0.00, I^2^ = 99.45%), -LR (*P* = 0.00, I^2^ = 98.13%) were all heterogeneous. The Bbivariate mixed-effects regression model was used for data merging. The results showed that the DOR was 18.19 (95% CI: 10.99 ~ 30.11) (Fig. 4a), sensitivity = 0.81 (95%CI: 0.75 ~ 0.87), specificity = 0.81 (95%CI: 0.72 ~ 0.87) (Fig. 4b), +LR = 4.2 (95%CI: 2.95 ~ 6.00), -LR = 0.23 (95%CI: 0.17 ~ 0.31) (Fig. 4c), and AUC was 0.88 (95%CI = 0.85 ~ 0.90). The Fagan’s Nomogram results showed that when the current test probability was 20%, the post-test probability of +LRwas 51%, and the post-test probability of Personality -LR was 5% (Fig. 4d). The Deeks’ funnel plotshowed *p* = 0.10, indicating no obvious publication bias (Fig. 5). The results of subgroup analysis of publication year, ethnic population, test sample size and cut-off value showed that (Fig. 6), the influence of publication year test sample size and cut-off value on sensitivity results was statistically significant. The effect of test sample size on specificity results was extremely significant (*P* < 0.01), indicating that these factors might be the main sources of heterogeneity. From the above results, HbA1cC is of good value in the diagnosis of diabetic retinopathy.

### Sensitivity analysis

**Fig. 7:**
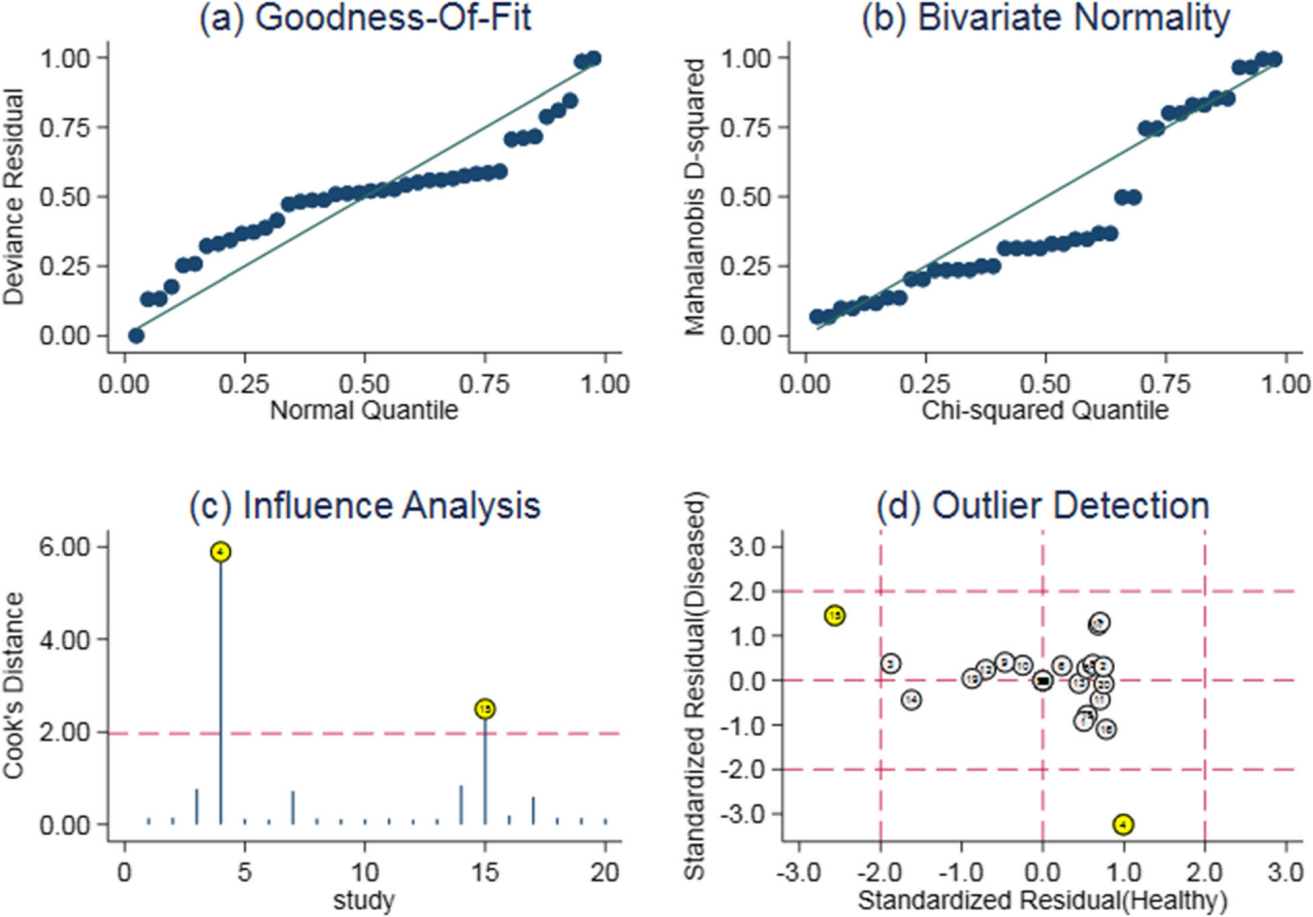
The results of sensitivity analysis (**a**: Goodness of fit. **b**: Bivariate normality. **c**: Influence analysis. **d**: Outlier detection)

The sensitivity analysis results of HbA1c diagnostic accuracy for diabetic retinopathy were shown in Fig. 7 (a,b,c,d). The Goodness-of-fit and bivariate normal analysis showed that the bivariate mixed-effects model was robust for meta-analysis. Furthermore, the robustness of the meta-analysis was determined by influence analysis and outlier detection analysis. After excluding outliers, there was no significant change in overall sensitivity (0.81 vs. 0.82), specificity (0.81 vs. 0.82), +LR(4.2 vs. 4.6), -LR(0.23 vs. 0.21), DOR (18.19 vs. 21) and AUC (0.88 vs. 0.88), which showed that the conclusions of the meta-analysis were robust.

## Discussion

Along with the increase in the number of diabetic patients and the increasing prevalence in various regions of the world, diabetic retinopathy has become one of the main eye diseases that cause blindness in China [36]. The Wiscnsin Epidmiologic Study of Diabetic retinopathy (WESDR), a world-renowned USA study, has reported that the incidence of DR is 50.1%, and the incidence of retinopathy is related to the duration of diabetes. That is, the longer the course of diabetes, the higher the incidence of retinopathy [37]. It has been reported that there is a significant correlation between the incidence of diabetic retinopathy and the level of HbA1c. The UKPDS study has shown that for patients with type 2 diabetes, their HbA1c levels are reduced by 1%, and the risk of retinopathy can be reduced by about 21% [38]. In a 2004 observation on retinal thickness and blood-retinal barrier in patients with type 2 diabetes [39], after 3 years of follow-up, it was found that high HbA1c levels were an independent risk factor for blood-retinal barrier. Therefore, attaining the standard of HbA1c can improve or delay the development of diabetic retinopathy. Patients with persistently high glycosylated hemoglobin levels have poor blood glucose control and a significant increase in basal metabolic rate, resulting in a significant increase in tissue oxygen demand, which makes their tissues often in the state of hypoxia. At the same time, there is a kind of glycosylated hemoglobin with high affinity to oxygen in erythrocytes, which can prevent hemoglobin from binding to 2-3DPG, which makes the oxygen not easy to dissociate, resulting in tissue hypoxia and the proliferation of vascular growth factor, which is the basis of the occurrence and progression of diabetic retinopathy [40]. It is also believed that the aggregation rate of red blood cells has a significant impact on the level of glycosylated hemoglobin. When the level of glycosylated hemoglobin in patients with diabetes is higher, a large number of red blood cells in the body will gather with each other at a faster speed, making fundus micro-vessels easy to form thrombus, which is the pathophysiological basis of early diabetic retinopathy. Su SC [41] and other studies have found that type 2 diabetic patients with higher HbA1c levels have a significantly higher prevalence rate of retinopathy than diabetic patients with normal HbA1c. And when HbA1c ≥ 7.0%, the incidence of retinopathy is about 85%. Studies have shown that there is no significant difference in the prevalence rate of diabetic retinopathy in diabetic patients with HbA1c of 6.0–8.0%, but the incidence of diabetic retinopathy in patients with HbA1c ≥ 8.0% is significantly higher than that in patients with HbA1c < 8.0% [42].

In order to obtain more accurate data about the diagnostic value of HbA1c for diabetic retinopathy, this meta-analysis was carried out. This study included 18 papers and a total of 2345 patients with diabetic retinopathy. The results showed that HbA1c is an effective indicator for the diagnosis of diabetic retinopathy. The overall sensitivity and specificity were 0.8, suggesting that the missed diagnosis rate and misdiagnosis rate of HbA1c in the diagnosis of diabetic retinopathy were both 19%. The area under the SROC curve is an index to measure the accuracy of the diagnostic test. The closer the area under the curve is to 1, the better the diagnostic effect is. In this study, the AUC was 0.88, indicating that HbA1c is more effective in the diagnosis of diabetic retinopathy. Sensitivity analysis showed that the results of this study did not change significantly with the elimination of a single study, indicating that the results of this study are relatively robust and reliable. Bias is the most important challenge in the process of Meta-analysis. The publication bias evaluation results of this study showed that *P* > 0.05, indicating that the results of this meta-analysis were integrated, comprehensive, objective and true revealing of the 20 studies included, and have high credibility and practical significance. The results are consistent with the results of a meta-analysis study conducted by Martínez-Vizcaíno et al. [43] in 2015, The research results of whom have shown that HbA1c and 2 h-PG have similar diagnostic accuracy, which is better than fasting blood-glucose (FBG) and 2-h post-meal blood glucose (2 h-PG) is always the first choice for clinical diagnosis of diabetes, but because of its time-consuming and laborious, HbA1c is considered to be a better substitute. Our study increased the number of studies included and the number of cases on his basis, and further verified and supported this conclusion.

This study also has certain limitations: 1) The number of included papers was small, only Chinese and English papers were available, while high-quality papers in other languages were likely to be missed. 2) there was great heterogeneity among the results of this study, and only some indicators were analyzed by subgroup analysis, which was not enough to well explore the source of heterogeneity. 3) Different studies had different diagnostic thresholds, which might result in bias in the results.

In conclusion, HbA1c has a good diagnostic valueeffectiveness for diabetic retinopathy and may be used for preliminary screening of clinical diabetic retinopathy. In future studies, the diagnostic effectiveness of HbA1c may be increased by adjusting the appropriate diagnostic threshold, or combing it with some other biochemical indicators such as FPG, 2 h-PG and other biochemical indicators to improve its diagnostic effectiveness. More clinical data are still needed to further verify the above conclusions.

## Data Availability

The datasets used and/or analysed during the current study are available from the corresponding author on reasonable request.

## Abbreviations

HbA1c: Glycosylated hemoglobin
DR: Diabetic retinopathy
AUC: Area under the curve
DM: Diabetes Mellitus
T2DM: type 2 diabetes mellitus
QUADAS-2: Quality assessment of diagnostic accuracy studies-2
SROC: Summary receiver operatingoperator characteristic
FBG: Fasting blood-glucose
2 h-PG: 2-h post-meal blood glucose
